# Evaluation of the effects of mulch on optimum sowing date and irrigation management of zero till wheat in central Punjab, India using APSIM

**DOI:** 10.1016/j.fcr.2016.08.016

**Published:** 2016-10

**Authors:** Balwinder-Singh, E. Humphreys, D.S. Gaydon, P.L. Eberbach

**Affiliations:** a International Maize and Wheat Improvement Center (CIMMYT), CG Block, National Agricultural Science Center (NASC) Complex, DPS Marg, New Delhi 110012, India; b Formerly; IRRI, Philippines; c Current address; Griffith, NSW 2680, Australia; d CSIRO Agriculture and Food, Queensland BioScience Precinct, St. Lucia Q 4067, Australia; e School of Agriculture and Food Sciences, University of Queensland, St. Lucia Q 4067, Australia; f Charles Sturt University, Wagga Wagga, NSW 2678, Australia

**Keywords:** North-western India, Water productivity, Yield, Soil evaporation, Soil type, Soil water deficit

## Abstract

Machinery for sowing wheat directly into rice residues has become more common in the rice-wheat systems of the north-west Indo-Gangetic Plains of South Asia, with increasing numbers of farmers now potentially able to access the benefits of residue retention. However, surface residue retention affects soil water and temperature dynamics, thus the optimum sowing date and irrigation management for a mulched crop may vary from those of a traditional non-mulched crop. Furthermore, the effects of sowing date and irrigation management are likely to vary with soil type and seasonal conditions. Therefore, a simulation study was conducted using the APSIM model and 40 years of weather data to evaluate the effects of mulch, sowing date and irrigation management and their interactions on wheat grain yield, irrigation requirement (I) and water productivity with respect to irrigation (WP_I_) and evapotranspiration (WP_ET_). The results suggest that the optimum wheat sowing date in central Punjab depends on both soil type and the presence or absence of mulch. On the sandy loam, with irrigation scheduled at 50% soil water deficit (SWD), the optimum sowing date was late October to early November for maximising yield, WP_I_ and WP_ET_. On the clay loam, the optimum date was about one week later. The effect of mulch on yield varied with seasonal conditions and sowing date. With irrigation at 50% SWD, mulching of wheat sown at the optimum time increased average yield by up to 0.5 t ha^−1^. The beneficial effect of mulch on yield increased to averages of 1.2–1.3 t ha^−1^ as sowing was advanced to 15 October. With irrigation at 50% SWD and 7 November sowing, mulch reduced the number of irrigations by one in almost 50% of years, a reduction of about 50 mm on the sandy loam and 60 mm on the clay loam. The reduction in irrigation amount was mainly due to reduced soil evaporation. Mulch reduced irrigation requirement by more as sowing was delayed, more so on the sandy loam than the clay loam soil. There was little effect of mulch on irrigation requirement for late October sowings.

There were large trade-offs between irrigation input, yield, WP_ET_ and WP_I_ on the sandy loam with regard to the optimum irrigation schedule. Maximum yield occurred with very frequent irrigation (10–20% SWD) which also had the greatest irrigation input, while WP_I_ was highest with least frequent irrigation (70% SWD), and WP_ET_ was highest with irrigation at 40–50% SWD. This was the case with and without mulch. On the clay loam, the trade-offs were not so pronounced, as maximum yield was reached with irrigation at 50% SWD, with and without mulch. However, both WP_ET_ and WP_I_ were maximum and irrigation input least at the lowest irrigation frequency (70% SWD). On both soils, maximum yield, WP_ET_ and WP_I_ were higher with mulch, while irrigation input was slightly lower, but mulch had very little effect on the irrigation thresholds at which each parameter was maximised.

## Highlights

• Late October to early November sowings gave maximum yield and irrigation WP of both mulched and non-mulched wheat in NW India.
• Mulch increased yield of late October-early November sowings, but decreased yield of later sowings.
• Mulch reduced the number of irrigations by one in about 50% of years under practical irrigation schedules (∼50% SWD).
• Maximum yield on sandy loam was at 10% SWD and at 10–50% SWD on clay loam, and least irrigation and highest WPI was at 70% SWD scheduling on both soils.
• Maximum WP_ET_ occurred with scheduling at 40–60% and 70% SWD on the sandy loam and clay loam, respectively.

## 1 Introduction

The highly mechanised, irrigated rice-wheat systems of north-west India (Punjab and Haryana states) are particularly important for food security in India as they contribute about 69% of the total food procurement of the Indian government. However, the sustainability of these systems is threatened by soil, water, nutrient and environmental issues (Ladha et al., 2007, Bijay-Singh et al., 2008). Almost all of the rice in this region is harvested by large combine harvesters, followed by in situ burning of the residues prior to sowing wheat. Residue incorporation is unattractive due to the cost and time taken, because many tillage passes are required, and because of N-immobilisation and the need to delay wheat sowing by several weeks to avoid N deficiency (Yadvinder-Singh et al., 2004). Burning quickly removes the residues, but causes serious air pollution and loss of organic matter and nutrients (Gupta et al., 2004, Yadvinder-Singh et al., 2005).

The recent development of the ‘Happy Seeder’ provides the capability of drilling wheat directly into the anchored and loose rice residues (Sidhu et al., 2007, Sidhu et al., 2008, Sidhu et al., 2015), avoiding the need to burn. The most recent version, the Turbo Happy Seeder, cuts and chops the straw in front of the sowing tynes, and deposits it as surface mulch between the seed rows. A major advantage of the technology is time saving between rice harvest and wheat sowing. Timely sowing reduces the risk of terminal heat stress during grain filling. However, mulch delays the anthesis of wheat sown at the recommended time by 7–10 d (Balwinder-Singh et al., 2011c), pushing grain filling into warmer weather. Several field experiments in the region showed that mulch maintains or increases the yield of wheat sown at the recommended time (Balwinder-Singh et al., 2011c, Chakraborty et al., 2008), and that it reduces irrigation water requirement in some years (Balwinder-Singh et al., 2011c, Naveen-Gupta et al., 2016, Yadvinder-Singh et al., 2008) due to conservation of soil moisture as a result of suppression of soil evaporation (Es) (Balwinder-Singh et al., 2011b). However, the effects of sowing date and mulch on irrigation requirement and other components of the water balance over the range of likely seasonal conditions are not known. Furthermore, the effect of mulch may vary with irrigation management, and also with soil type because of differences in properties such as plant available soil water capacity and hydraulic conductivity.

Rainfall (long term average 120 mm) during the wheat season in Punjab does not meet the crop water (evapotranspiration, ET) needs (∼400 mm). Furthermore, temporal rainfall distribution is usually poor in relation to crop requirement. Hence, there is need for irrigation to achieve high yield in most years. Farmers usually apply 4–5 irrigations during the wheat season, and in the rice-wheat areas groundwater is the main source of irrigation water (Ambast et al., 2006). However, groundwater depletion is a serious threat to the sustainability of rice-wheat systems in the region (Humphreys et al., 2010). Therefore, there is a need to identify management options that maximise crop water productivity (WP_ET_), and this requires an understanding of the impacts of mulch over the likely range of seasonal and site conditions.

Well-tested crop models can be useful tools to extrapolate results from site specific studies conducted in a limited number of seasons, management and environments to other situations and, using historical climate data, a much longer time period. The results can be used to identify the optimum management practices, which may vary depending on the objectives of the farmer or water resource manager. In the past, various crop models have been used in north-west India for a range of purposes including determination of: the potential yield of rice and wheat (Pathak et al., 2003); yield gaps in rice and wheat crops (Aggarwal et al., 2000); the effects of climate change on yield of rice and wheat (Pathak and Wassmann, 2009); the effects of irrigation scheduling and sowing date on yield and water productivity of wheat (Timsina et al., 2008); the interaction between irrigation and nitrogen management on wheat yield (Arora et al., 2007); and the irrigation requirement and water productivity of rice-wheat and alternative cropping systems (Jalota and Arora, 2002). However, to date, crop models have not been used to evaluate the effects of mulching wheat on optimum sowing date and irrigation requirement, either in north-west India or globally. Therefore the aims of the work presented here were to use the APSIM model (Holzworth et al., 2014) to determine: 1) the effect of mulch on the optimum sowing date of irrigated wheat in north-west India, 2) the optimum irrigation management of wheat, as affected by soil type and mulch, and 3) the impacts of mulch on yield, irrigation water requirement, components of the water balance, and various measures of water productivity.

## 2 Methods

### 2.1 APSIM model (v. 7.6)

APSIM is a simulation modelling framework that enables sub-models to be linked to simulate agricultural system performance. In simulating wheat cropping, the four modules used are Wheat, Soilwat, SoilN and SurfaceOM. The Wheat module simulates crop development, growth, water and N uptake, crop N concentration, stresses (water deficit, N deficit, aeration deficit) and the response of the crop to the stresses (Keating et al., 2003). The Wheat module is based on CERES Wheat (Jones and Kiniry, 1986; Ritchie et al., 1985) but with modifications (Asseng et al., 1998, Probert et al., 1995, Wang et al., 2003). Soilwat is a cascading water balance model based on the water balance models in the CERES and PERFECT models. SoilN is based on the CERES model (Ritchie et al., 1985), with modifications (Probert et al., 1998). The surface organic matter module was developed by Probert et al. (1995) and is described in detail by Thorburn et al. (2001).

### 2.2 Simulations

The locally calibrated and validated APSIM-Wheat model (Balwinder-Singh et al., 2011a, Balwinder-Singh et al., 2015) was used to study the effects of sowing date on yield, components of the water balance and water productivity of irrigated wheat at Ludhiana, Punjab. The model was then used to study the effects of mulch and irrigation schedule, and their interactions, for the optimum sowing date on two soil types (clay loam and sandy loam).

The model was calibrated for wheat variety PBW343, with and without mulch separately, as field data showed that mulch delayed anthesis by 6–8 days, probably due to reduced soil temperature (Balwinder-Singh et al., 2011c). This effect of mulch on soil temperature and thus crop development is not captured by the model (Balwinder-Singh et al., 2011a). The values of the coefficients for wheat cv. PBW343 grown without mulch were: startgf_to_mat (grain filling duration in degree days, °C) = 750, tt_floral_initiation (degree days to start anthesis, °C) = 400, vern_sens (sensitivity to vernalisation) = 1.7, photop_sens (photoperiod sensitivity) = 3.8. The values for all coefficients for the mulched crop were the same as for the non-mulched crop except for ‘tt_floral_initiation’ = 450 to capture the delayed anthesis under mulched conditions. We also manually modified the wheat phenology response to temperature, employing a trapezoidal response curve rather than a triangular response curve from the standard APSIM release (for details, see Balwinder-Singh et al., 2015) to capture the appropriate effect of high temperature on crop phenology. In all simulation scenarios, the initial conditions were set on 15 October (a typical rice harvest date in Punjab) with soil water content at 80% of field capacity in the top 30 cm, and at field capacity below this depth, reflecting the wet soil profile following ponded rice. The initial total available soil water in the 0–180 cm profile was 316 and 273 mm in the clay loam and sandy loam soils, respectively. There was no tillage prior to sowing, and a plant density of 150 m^−2^ was used with row spacing 20 cm, sowing depth 5 cm, and non-limiting nutrients. The simulations were performed over a 40 year period (1970–2010) using daily weather data from the meteorological station at Punjab Agricultural University (PAU), Ludhiana. Average or total monthly data for the wheat season are presented in Table 1. In the mulched treatments, the initial conditions included application of 8 t ha^−1^ of rice straw mulch on 15 October. The non-mulched treatments had a bare soil surface, to represent the practice of removal of straw by burning after rice harvest, prior to establishment of wheat. In the sowing date simulations (Scenarios 1 and 2), irrigations were scheduled whenever the soil water deficit (SWD) of the 0–60 cm soil profile increased to 50%, and available water in the same soil profile (0–60 cm) was also used for the irrigation scheduling simulations (Scenario 3). The amount of irrigation water applied was 120% of SWD (0–60 cm) to represent the inherent inefficiency of flood irrigation.

All simulations were performed using PBW 343 for two soil types, sandy loam and clay loam (Table 2), to represent the range in major soil types used for rice-wheat systems in Punjab. The soil parameters were based on the properties of field sites at Punjab Agricultural University, Ludhiana with sandy loam (Timsina et al., 2008, Yadvinder-Singh et al., 2009) and clay loam (Balwinder-Singh et al., 2011c). The sandy loam and clay loam soils had a plant available water capacity (PAWC) of 110 and 128 mm, respectively, over the 0–60 cm soil profile, and PAWC of 290 and 335 mm over the 0–180 cm soil profile. The stage 1 soil evaporation parameter (U) was set to 10 mm for the sandy loam based on the values used by Arora et al. (2007) and Timsina et al. (2008), and 12 mm for the clay loam soil based on the results of Balwinder-Singh et al. (2011b). The Es stage 2 parameter (cona) was set to 2 and 4 mm for the sandy loam and clay loam, respectively, based on the above studies. The initial soil mineral N (0–150 cm soil depth) was set to 120 kg N ha^−1^, representing typical soil mineral N content prior to wheat establishment in rice-wheat systems in this region (Arora et al., 2007).

The results of the simulations were analysed in terms of grain yield (dry), components of the water balance, and water productivity. The components of the water balance examined were irrigation amount, Es, transpiration, ET, deep drainage beyond 180 cm depth, and runoff. Water productivity was computed with respect to ET (WP_ET_) and irrigation (WP_I_).

WP_ET_ (kg ha^−1^ mm^−1^) = Grain yield (kg ha^−1^)/Total seasonal ET (mm)

WP_I_ (kg ha^−1^ mm^−1^) = Grain yield (kg ha^−1^)/Total irrigation amount (mm)

The water stress index was used to compare the severity of water deficit as affected by soil type. This index is a factor (swdef_photo) used to modify the amount of photosynthesis, and is calculated daily in APSIM. Values range from 1 to 0, where 1 = no stress and 0 = maximum stress.

#### 2.2.1 Scenario 1—effect of sowing date

APSIM was used to assess the climatically determined (i.e. no water and nutrient stress) potential yield of PBW 343 for nine sowing dates from 10 October to 30 December, at 10-day increments. The simulations were performed both using non-limiting water, and also under realistic conditions with irrigation scheduled when SWD increased to 50% of PAWC (0–60 cm), for sandy loam and clay loam soils. The objective was to determine the optimum sowing date for well-irrigated wheat taking into account the trade-offs between yield, irrigation amount, WP_I_ and WP_ET_.

The photothermal quotient (PTQ) (Ortiz-monasterio et al., 1994), an index of growth per unit development time which assumes that development rate is linearly related to mean temperature, was also calculated for the period from maximum tillering to anthesis for the maximum and minimum yielding years using the formulae:

If T ≥ 10 then PTQ day^−1^ = solar radiation/(T − 4.5)

If 4.5 ≤ T ≤ 10 then PTQ day^−1^ = solar radiation × [(T − 4.5)/5.5]/5.5

If T ≤ 4.5 then PTQ day ^−1^ = 0

where T is the daily mean temperature in °C and PTQ is expressed as MJ m^−2^ day^−1^ °C^−1^

#### 2.2.2 Scenario 2—effect of mulch x sowing date

The interactions between mulch and sowing date on the performance of well-irrigated wheat were studied for seven sowings from mid-October to late November with an increment of 7–8 days between sowings. The objective was to determine whether mulch influences the optimum sowing date.

#### 2.2.3 Scenario 3—effect of irrigation schedule x mulch

Seven treatments with irrigations scheduled according to SWD and rainfed wheat were compared. Irrigations were applied when SWD reached 10%, 20%, 30%, 40%, 50%, 60% and 70% of PAWC. The crop was sown on 7 November − within the optimum sowing window for this region. The objective was to determine the effects of mulch on irrigation requirement, and trade-offs between irrigation amount, yield and water productivity.

### 2.3 Statistical analysis

The data were analysed by analysis of variance (ANOVA) using Genstat (v 13.0) with a factorial design keeping *residue* and *sowing dates* as factors and *years* as replicates. The differences between treatments were evaluated for their significance using the least significant difference (LSD) at the 95% confidence level.

## 3 Results

### 3.1 Scenario 1—optimum sowing date

**Fig. 1:**
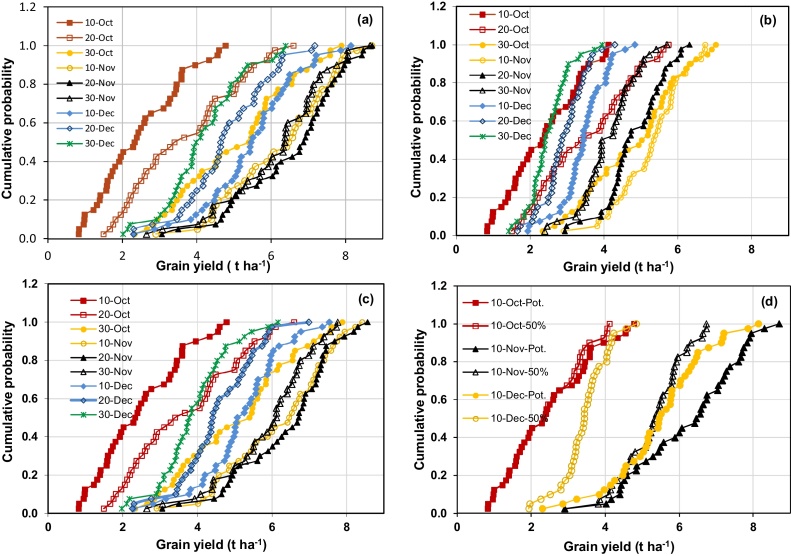
Effect of sowing date on (a) potential grain yield of wheat, (b) grain yield with irrigation scheduled at 50% SWD on a sandy loam, (c) grain yield with irrigation scheduled at 50% SWD on a clay loam soil, (d) potential yield and grain yield at 50%SWD on a sandy loam (Scenario 1).

Potential grain yield was strongly affected by sowing date and by seasonal weather conditions (Fig. 1a). For example, with sowing on 10 November, potential yield ranged from 3.0 to 8.5 t ha^−1^ over the 40 years. Potential yield was usually highest with 20 November sowing (mean 6.4 t ha^−1^), closely followed by 10 November (mean 6.3 t ha^−1^) sowing (Table 3). Potential yield increased as sowing date was delayed from 10 October (mean 2.9 t ha^−1^) to 20 November, and then declined with delay in sowing beyond that. Average potential yield decreased by 52 kg ha^−1^ day^−1^ (0.8% d^−1^) with delay in sowing from 10 November to 30 December.

With irrigation at 50% SWD, yield was again strongly affected by both sowing date and seasonal conditions on both soils (Fig. 1b, c). However, the effect of sowing date varied somewhat from that of potential yield. In particular, the optimum sowing date for maximum yield on the sandy loam was earlier (10 November, mean 5.6 t ha^−1^), followed by 30 October (mean 5.2 t ha^−1^). For each sowing date, yield with 50% SWD scheduling on the sandy loam was always lower than potential yield (Fig. 1d), while yields on the clay loam were generally similar (Table 3). For example, average yield on the sandy loam (5.6 t ha^−1^) was 17% lower than average potential yield (6.3 t ha^−1^) for 10 November sowing. The size of the difference increased as sowing was delayed. This was due to increasing soil water deficit stress with irrigation scheduled at 50% SWD, more so on the sandy loam. For example, in 1997, potential yield of the 10 November sowing was 6.6 t ha^−1^, compared with yields of 5.8 and 6.5 t ha^−1^ on the sandy loam and clay loam soils respectively, while water deficit stress was small to negligible on both soils (average water stress indices over the whole season of 0.94 and 0.99, respectively). In the same year, yield of the 30 November sowing was 4.8 and 6.5 t ha^−1^, respectively, with water stress indices of 0.90 and 0.99.

The rate of decline in yield with delay in sowing was higher on the sandy loam (50% SWD irrigation scheduling) than for potential yield. Yield with scheduling at 50% SWD decreased by an average of 62 kg ha^−1^ day^−1^ (1.1% d^−1^) when sowing was delayed from 10 November to 30 December on the sandy loam, compared with 52 kg ha^−1^ d^−1^ or 0.8% d^−1^ on the clay loam. With irrigation scheduling at 50% SWD, average irrigation amount was least for sowings on 10 October on both soils, while average WP_ET_ and WP_I_ were highest for sowings from late October to early November on both soils (Table 3). Average WP_I_ was much higher on the clay loam than on the sandy loam for all sowing dates. There were trade-offs between minimising irrigation input and maximising yield, WP_I_ and WP_ET_ on both soils. While irrigation input was least for sowing on 10 October, maximum yield, WP_I_ and WP_ET_ occurred for various sowing dates from 30 October to 20 November depending on soil type and parameter.

### 3.2 Scenario 2—effect of mulch on optimum sowing date

**Fig. 2:**
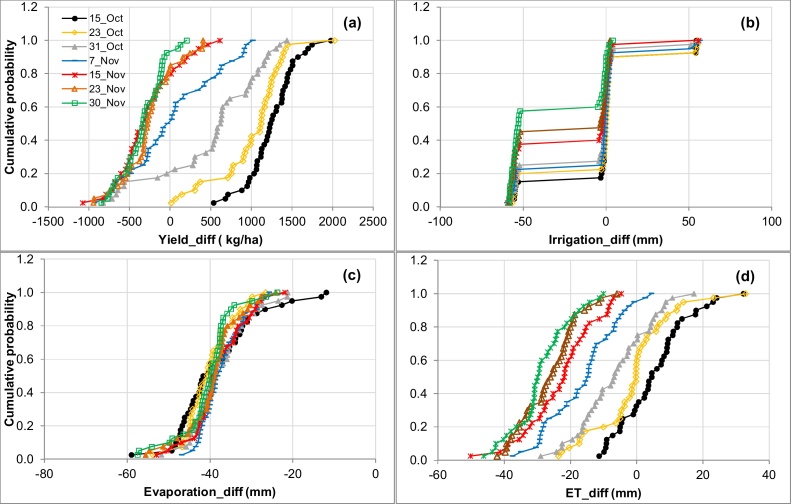
Effect of sowing date on probability of difference (mulch minus non-mulch) in (a) grain yield, (b) irrigation water input, (c) soil evaporation, and (d) evapotranspiration (ET) on the sandy loam soil with irrigation scheduled at 50% SWD (Scenario 2).

There were significant (P < 0.05) interactions between sowing date and mulch treatments on grain yield, ET and amount of irrigation on the sandy loam soil. The earliest sowings (15 and 23 October) always had higher yield with mulch than without mulch and by larger amounts with 15 October sowing (Fig. 2a). Mulch resulted in yield loss with increasing frequency and severity as sowing was delayed up to 30 November. For example, the effect of mulch on yield of 23 October sowings ranged from +10 to +2000 kg ha^−1^ (mean 1000 kg ha^−1^), compared with −850 to +200 kg ha^−1^ (mean −325 kg ha^−1^) for 30 November sowings (Table 4). Mulch reduced yield in 20% and 90% of years for the 31 October and 30 November sowings, respectively. On the clay loam soil, similar trends were observed; however, the frequency and severity of yield loss within sowing date was less than on the sandy loam (data not presented). For example, there was no yield loss with mulch on the clay loam soil for the 31 October sowing in any year. Mean yield advantage with mulch was always higher (or mean yield loss always lower) on the clay loam than on the sandy loam, except for the 15 October sowing (Table 4). However, mean yield loss for the sowings on 23 and 30 November was only slightly lower on the clay loam than the sandy loam. The optimum sowing date window for mulched wheat with irrigation at 50% SWD was 31 October-7 November on the sandy loam, compared with 7–14 November on the clay loam.

Mulch had no effect or decreased irrigation requirement in most years for mid to late November sowings on the sandy loam soil (Fig. 2b). For the 7, 15, 23 and 30 November sowings, one less irrigation (roughly 55 mm) was required with mulch in 25, 40, 45 and 60% of years, respectively. However, for all October sowings, mulch reduced the number of irrigations in less than 20% of years, and resulted in one additional irrigation in 2–4 out of 40 years, due to longer crop duration. On the clay loam, the effect of mulch on irrigation frequency and amount was smaller (data not presented). For example, with 15 November sowing, mulch reduced the irrigation amount in 30% of years on the clay loam, compared to 40% of years on the sandy loam, and the difference increased with delay in sowing. With 30 November sowing, mulch reduced the irrigation amount in 38% of years on the clay loam, compared to 60% of years on the sandy loam soil.

Mulch suppressed Es, usually by 20–60 mm, and by means of around 40 mm, on the sandy loam soil. The effect of mulch on Es was similar (usually within 10 mm) for all sowing dates (Fig. 2c). The effect of mulch on suppression of Es was slightly higher on the clay loam (by means of around 45 mm, range 25–74 mm) than on the sandy loam soil. Mulch generally increased transpiration (T) in all years by 2–3 to 61–70 mm on both soils across all sowing dates (data not presented). Late October sowings had the biggest increase in T with mulch.

The effect of mulch on ET was relatively small on both soils as a result of opposing effects on Es and T, and varied from a mean decrease of 30 mm to a mean increase of 5 mm across sowing dates on the sandy loam (Fig. 2d) and clay loam soils (data not presented). Mulch decreased ET more as sowing was delayed, and by slightly more on the clay loam (Table 4). For example, mulch decreased ET in 35% of years for the 15 October sowing, and in all years for the 15, 23 and 30 November sowings (by from 6 to 50 mm) on the sandy loam soil. On the clay loam, mulch decreased ET in 50% of years for the 15 October sowing and in all years for the 15, 23 and 30 November sowings.

On the sandy loam, average yield, WP_I_ and WP_ET_ of non-mulched wheat were all maximised with sowing on 31 October to 7 November, while with mulch all were maximised by sowing on 23 October. On the clay loam without mulch, all were maximised with sowing on 7 November. However, in the presence of mulch, there were small trade offs between maximising yield and WP_ET_ (31 October sowing) and maximising WP_I_ (23 October sowing).

### 3.3 Scenario 3—effect of mulch on optimum irrigation scheduling

#### 3.3.1 Irrigation amount and number

The number of irrigations was strongly affected by irrigation schedule on both soils. For 7 November sowing, averages of 24–27 irrigations and application rates of 15–20 mm per irrigation were required when irrigating at 10% SWD, compared with averages of 2 irrigations of 70–120 mm at 70% SWD (Table 5). Clearly, irrigation at 10–20% SWD is not practical for flood irrigated wheat, but it is representative of the possibilities with sprinkler or drip irrigation. An irrigation schedule of 40–50% SWD (average 4–6 irrigations, 50–70 mm per irrigation) is probably more typical of farmer practice.

**Fig. 3:**
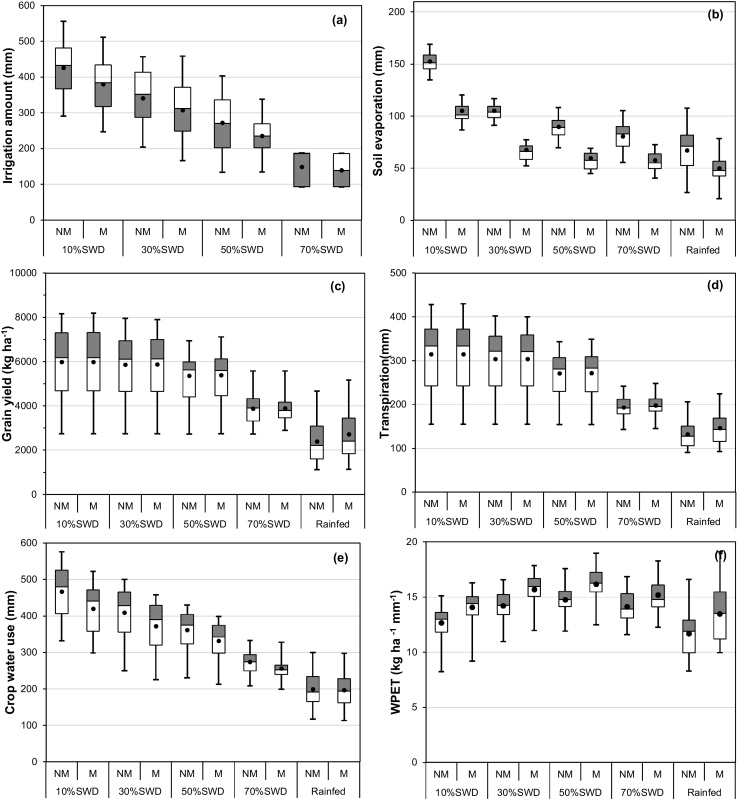
Effect of irrigation and residue treatments on simulated (a) irrigation amount, (b) grain yield, (c) transpiration, (d) soil evaporation, (e) crop water use (ET), (f) water productivity (WP_ET_), on sandy loam soil over 40 years. Error bars represents range, (■) represents 75th percentile, (▭) represents 25th percentile, (●) represents average values. NM = non-mulch, M = mulch (Scenario 3).

Within irrigation treatment, the total amount of irrigation water applied varied greatly with seasonal conditions (Fig. 3a). For example, in non-mulched wheat sown on 7 November on the sandy loam and irrigated at SWD 50%, the number of irrigations ranged from 2 to 6, while the amount applied ranged from 133 to 403 mm. The lowest amount occurred in a higher rainfall year (194 mm in 1991–1992) when rainfall was more evenly distributed during the crop growth period than in other years with similar total in-season rainfall. The highest irrigation amount occurred in a year of low (41 mm, 1975) rainfall which fell in a few small events. The mean number of irrigations and total irrigation amount increased as the threshold for irrigation decreased from 70 to 10% SWD on both soils, with and without mulch (Fig. 3a, Table 5). Within irrigation schedule and mulching treatment, the average number of irrigations was similar on both soils. However, the amount of irrigation was higher on the clay loam than the sandy loam, more so with infrequent irrigation (SWD 60 and 70%).

Mulch reduced the amount of irrigation required, more so as the irrigation threshold decreased, and more so in drier years (Fig. 3a, Table 5). For example, with irrigation at 10% SWD, mulch reduced the average amount of irrigation by 55 and 82 mm on the sandy loam and clay loam soils, respectively, compared with reductions of 11 and 28 mm at 70% SWD. With irrigation at 50% SWD, mulch reduced the number of irrigations by one in almost 50% of years on both soils, a reduction of about 50 mm on the sandy loam and 60 mm on the clay loam. The reduction in irrigation with mulching was associated with lower Es (Fig. 3b).

#### 3.3.2 Grain yield

There was a large effect of irrigation schedule on grain yield on both soils, with and without mulch, however the response to irrigation schedule was greater on the sandy loam (Table 5). On the sandy loam, yield increased with increase in irrigation frequency from rainfed (mean yield 2.4 and 2.7 t ha^−1^ without and with mulch, respectively) to SWD 10% (mean 6.0 and 6.4 t ha^−1^) (Fig. 3c, Table 5). On the clay loam, average yield under rainfed conditions (3.2 and 3.5 t ha^−1^) was about 1.0 t ha^−1^ higher than on the sandy loam due to the higher PAWC of the clay loam. Yield on the clay loam increased with increasing irrigation frequency up to 50% SWD (6.0 and 6.5 t ha^−1^), with no change in yield at higher frequencies. Yield on the sandy loam increased by 39% when irrigation frequency increased from 70 to 10% SWD, compared with an increase of only 7% on the clay loam.

Mulch increased average grain yield by 0.1–0.5 t ha^−1^. On the sandy loam, the effect of mulch on average yield was greatest with irrigation scheduling at 20 and 30% SWD. On the clay loam, mulch increased average yield by 0.5 t ha^−1^ in all irrigation treatments except 70% SWD. The effect of mulch on rainfed yield was negligible in low yielding (dry) years, but increased to 0.8 and 1.0 t ha^−1^ in higher yielding (wetter) years on the sandy loam and clay loam, respectively.

#### 3.3.3 Water losses from the root zone

Soil evaporation increased with irrigation frequency (Fig. 3b, Table 5). For example, on the sandy loam, average Es increased from 67 mm in the rainfed treatment to 152 mm in the 10% SWD irrigation treatment without mulch. Average Es in the non-mulched treatments was higher on the clay loam than on the sandy loam, more so in the most frequently irrigated treatments.

The higher grain yield with more frequent irrigation was associated with higher transpiration (T) (Fig. 3d, Table 5). On the sandy loam, T of non-mulched wheat decreased from an average of 315–132 mm when irrigation frequency decreased from 10% SWD to rainfed, but there was only a small effect of irrigation frequency on T on the clay loam. Mulch increased T by averages of 18–30 mm, consistent with the improved crop growth (data not presented) and yield.

Without mulch, ET increased from 199 to 467 mm on the sandy loam, and from 250 to 506 mm on the clay loam, as irrigation frequency increased from rainfed to 10% SWD (Table 5). Mulch generally suppressed ET by 5%, and thus by a larger absolute amount in the more frequently irrigated treatments (Fig. 3e), and by more on the clay loam than on the sandy loam. The reduction in crop water use (ET) with mulch was due to the reduction in Es which more than offset the increase in T, with higher reduction in ET as irrigation frequency increased (Fig. 3e).

**Fig. 4:**
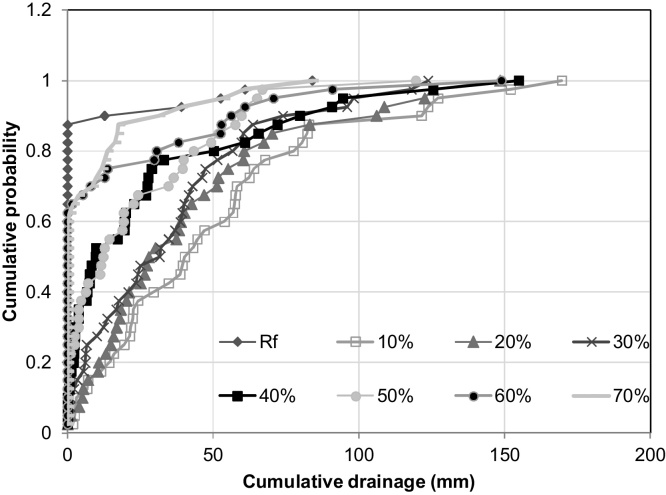
Effect of irrigation schedule on simulated deep drainage on sandy loam soil using 40 years weather data (Scenario 3). (Rf-rainfed, % = soil water deficit (SWD) irrigation trigger.

Deep drainage increased with irrigation frequency on both soils (Fig. 4, Table 5), with averages ranging from less than 10 mm in rainfed wheat to about 50 mm with irrigation at 10% SWD. Heavy rainfall events in some years resulted in large amounts of deep drainage. For example, in the 1997–1998 wheat season there was 102 mm of rain on 8 December which resulted in 83 mm of deep drainage in the rainfed treatment on the sandy loam soil, and amounts ranging to170 mm in the irrigated treatments depending on how recently the crop had been irrigated prior to the rain. There was a consistent trend for very slightly higher deep drainage with mulch, reflecting the slightly wetter soil conditions as a result of reduced Es.

There was no runoff in any treatment—the maximum rainfall of 102 mm on a single day was insufficient to generate runoff with a bund height of 100 mm.

Under rainfed conditions, there was large net depletion of water from the soil profile between sowing and harvest, by averages of 96 and 150 mm on the sandy loam and clay loam, respectively (Table 5). Soil water depletion decreased as irrigation frequency increased, with slight net wetting of the profile, on average, for the 10 and 20% SWD treatments on both soils. On average there was higher water depletion of the clay loam soil, especially in the rainfed and less frequently irrigated treatments. Mulch had a negligible effect on soil water depletion between sowing and harvest.

The effects of irrigation schedule and mulch on WP_ET_ were relatively small (Fig. 3f, Table 5). On the sandy loam, WP_ET_ peaked with irrigation at 40–60% SWD, with and without mulch. However, on the clay loam, WP_ET_ increased with decreasing irrigation frequency from 10 to 70% SWD. Within irrigation treatment, WP_ET_ was higher (by around 10%) with mulch due to both higher grain yield and lower ET. On both soils, irrigation water productivity (WP_I_) was strongly affected by irrigation schedule, and increased as irrigation frequency decreased due to a larger reduction in irrigation input than grain yield. Mulch increased WP_I_ by averages of 8–13% across irrigation schedules and soil types, due to both higher yield and reduced irrigation amount.

There were large trade-offs between irrigation input, yield, WP_ET_ and WP_I_ on the sandy loam with regard to the optimum irrigation schedule. Maximum yield occurred with very frequent irrigation (10–20% SWD) which required the greatest irrigation input, while WP_I_ was highest with least frequent irrigation, and WP_ET_ was highest with irrigation at 40–50% SWD. This was the case with and without mulch. On the clay loam, the trade-offs were not so pronounced, as maximum yield was reached with irrigation at 50% SWD, with and without mulch. However, both WP_ET_ and WP_I_ were maximum and irrigation input least at the lowest irrigation frequency (70% SWD). On both soils, maximum yield, WP_ET_ and WP_I_ were higher with mulch, while irrigation input was slightly lower, but mulch had very little effect on the irrigation thresholds at which each parameter was maximised.

## 4 Discussion

### 4.1 Effect of seasonal conditions on yield variability

The large variability in potential grain yield across the years was due to variability in climate. For example, for 10 November sowing, the highest potential yield (8.5 t ha^−1^ in 1988–1989) was associated with high solar radiation from the maximum tillering stage to anthesis (Table 6), consistent with findings of Fischer (2007) observed that solar radiation during the 15–20 d period before anthesis is important for biomass production and potential grain number. The PTQ (photothermal quotient) from maximum tillering to anthesis was also high in 1988–89. Fischer (1985) demonstrated that the number of grains m^−2^ (‘grain number’) increased with PTQ in normal sowings. The high yield in 1988–89 was also associated with high solar radiation during the grain filling period, while temperatures were average during this period. The lowest potential yield (3.0 t ha^−1^ in 1976–1977) was associated with low solar radiation during maximum tillering to anthesis, and hence low biomass production during the vegetative phase and low spike biomass at anthesis, and very low grain number (5700) compared to the average of 12,780 over 40 years. Maximum temperature was also relatively high during the grain filling period

### 4.2 Effect of sowing date on crop growth and yield

**Fig. 5:**
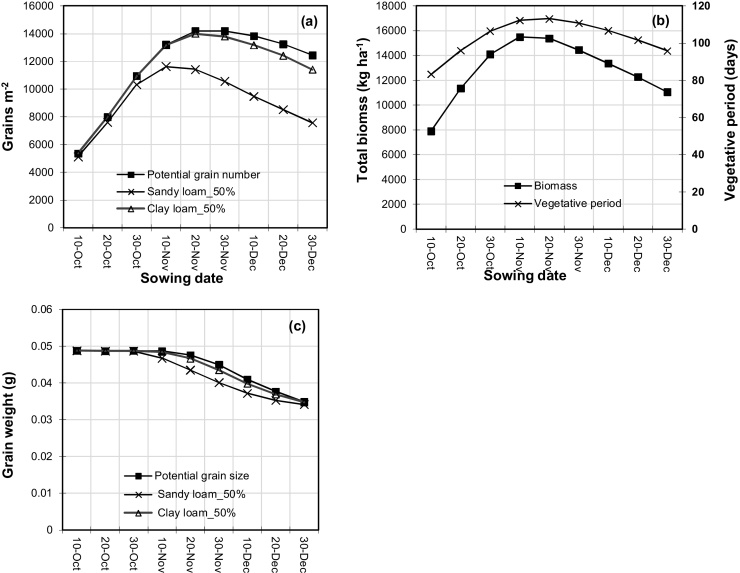
(a) Number of grains m^−2^ at 50% SWD irrigation as affected by sowing date in comparison with potential number, (b) total biomass and length of vegetative period for different sowing dates under potential yield conditions, and (c) grain weight at 50% SWD irrigation as affected by sowing dates in comparison with potential number (Scenario 1).

Maximum grain yield without mulch occurred with sowing on 7–15 November on the clay loam, and slightly earlier (30 Oct–7 November) on the sandy loam when irrigations were scheduled at 50% SWD (Table 4). The results on the clay loam are consistent with the field studies of Ortiz-monasterio et al. (1994) which showed that the optimum sowing date of similar duration varieties was 15 November for maximum yield at Ludhiana, and that grain yield decreased by about 0.8% per day delay in sowing beyond this date. Similarly, Randhawa et al. (1981) found yield declines of 0.9–1.2% per day delay in sowing beyond 25 October to 15 December. The lower grain yield of early sowings was associated with lower grain number (Fig. 5a) due to a shorter vegetative growth period, and lower LAI (data not presented) and biomass production (Fig. 5b). The lower grain yield with later sowing was associated with both lower grain number (especially on the sandy loam) and lower grain weight (Fig. 5c), as the grain filling period occurred during increasingly hot weather as sowing was delayed. There was little to no water deficit stress during the grain filling period for all sowing dates from 10 November to 30 December, with mean water stress index decreasing only slightly from 0.94 to 0.92 (sandy loam) and 0.99–0.97 (clay loam) as sowing was delayed. Similarly, the modelling study of Arora and Gajri (1998) found that the low yield of early sowings (early October) was associated with a shorter vegetative period and low grain number, while the low yield of late sowings was associated with shorter vegetative and reproductive periods.

The duration of phenological stages varied with sowing date in our simulation study. The duration of sowing to anthesis was longest for sowings on November 10 and 20, consistent with the findings of Ortiz-monasterio et al. (1994).

The results of our simulations of the effect of sowing date on potential yield using APSIM were also consistent with many of the findings of simulations using other crop models in this environment (Aggarwal et al., 2000, Arora et al., 2007, Arora and Gajri, 1998, Timsina et al., 2008), but there were also some differences, in terms of the magnitude of potential yield across sowing dates. The variable results across modelling studies point to the need for systematic comparative studies using common data sets to understand reasons for variation in model performance, promote continuous improvement, generate confidence limits in simulation output, and identify the best models for use in particular applications. Accurate assessment of potential yield is important because of its usefulness in determining yield gaps and evaluating options for closing yield gaps, and for identifying priorities for investment in research and development to reduce the gaps. Accurate simulation of water fluxes is important for the identification of options to increase water productivity and shift towards more sustainable cropping systems with regard to water depletion.

### 4.3 Effect of mulch x sowing date on yield and water balance

**Fig. 6:**
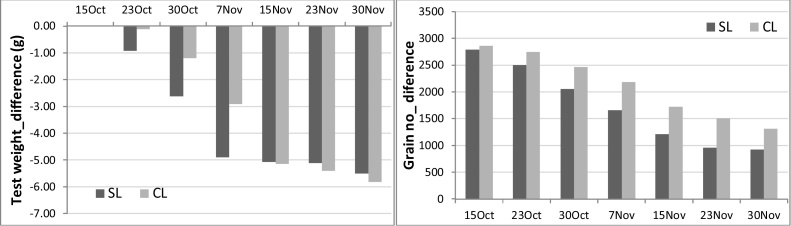
Effect of sowing date on the difference (mulch minus non-mulch) in (a) 1000 grain weight (test weight), and (b) grain number for wheat grown on sandy loam (SL) and clay loam (CL) soils (Scenario 2).

Our results showed a significant (P < 0.05) interaction between mulch and sowing date on yield, in contrast with the two-year field studies of Sidhu et al. (2007). This illustrates how modelling, in conjunction with short-term field experiments, can provide greater insights into long-term system performance and variability than field trials alone. In particular, our simulations showed that, with mulch, sowing could be brought forward as early as 23 October without yield loss on the sandy loam, whereas there was significant (mean 0.6 t ha^−1^) yield loss in the absence of mulch. Similarly, on the clay loam, sowing could be brought forward to 30 October without yield loss in the mulched wheat, while there was significant yield loss in the non-mulched wheat compared to 7–14 November sowing. On the other hand, when sowing was delayed to mid (sandy loam) or late (clay loam) November, mulch resulted in significant (mean 0.2–0.3 t ha^−1^) yield loss. Further evaluation of the interaction between mulch and sowing date is needed, using both field and simulation experiments, because of the practical importance of knowing how early wheat can be sown without yield loss, and whether, or under what circumstances, mulching of late sown wheat is detrimental to yield. In the simulations, the reduction in grain yield with mulch in late sown wheat was due to reduction in grain weight (Fig. 6a), which more than offset the higher grain number with mulch (Fig. 6b), which in turn was a result of higher biomass at anthesis.

**Fig. 7:**
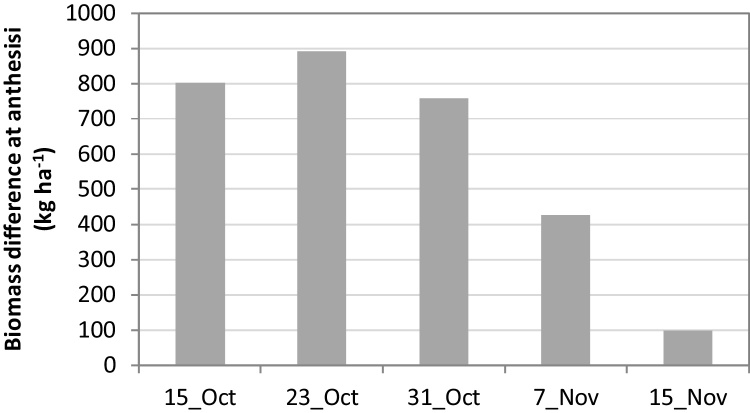
The difference (mulch minus non-mulch) in biomass at anthesis stage under different sowing dates (Scenario 2).

Potential grain number in APSIM Wheat is based on biomass at anthesis, and the increase in biomass at anthesis with mulch was highest for mid to late October sowings (Fig. 7) as a result of a longer vegetative phase and relatively higher temperature during the vegetative phase. As sowing was delayed beyond mid-October, the effect of mulch on crop duration decreased. The greater reduction in grain weight with mulch as sowing was delayed was due to exposure to higher temperature during the grain filling period of the mulched crops. High temperature during this period slows the rate of grain filling and accelerates senescence due to decrease in photosynthetic activities per unit leaf area (Al-Khatib and Paulsen, 1984, Zhao et al., 2007). Average temperature during the grain filling period was around 1.1 °C higher in mulched than non-mulched crops (for all sowing dates), but the effect was more damaging for later sowings due to the higher prevailing temperature, and in particular due to the higher number of days during which the crops were exposed to extreme temperatures. For example, for 23 October and 15 November sowings, average temperature during the grain filling period was 23 and 29 °C, respectively. Optimum temperature during the grain filling period for wheat is considered between 19.3–22.1 °C and temperature above 33.4 °C is considered to be damaging (Porter and Gawith, 1999). In APSIM, temperature above 26 °C decreases radiation use efficiency and temperature >34 °C accelerates senescence, which further shortens the grain filling period and also reduces the grain filling rate. As sowing was delayed from 15 October to 7 November, the number of days during which the crops were exposed to maximum daily temperature >34 °C during the grain filling period increased (Table 7). Within sowing date, the total number of days and thus the probability of exposure to high temperature during grain filling period were higher with mulch than without mulch. For example, with 7 November sowing, the mulched crop was exposed to high temperature during grain filling period on 185 d, compared to 111 d for the non-mulched crop during 40 crop seasons (Table 7).

There was a trade-off between the effect of mulch on yield and irrigation water savings as sowing was delayed. For example, with 30 November sowing on the sandy loam soil, mulch reduced irrigation amount by more than 50 mm in 60% of years compared to 20% of years for 15 October sowing, but average yield was also reduced by 0.3 t ha^−1^ by mulch under 30 November sowing compared with a 1.2 t ha^−1^ increase with mulch for 15 October sowing. However, on the clay loam soil, there was lower probability of mulch reducing irrigation amount with delay in sowing. For example, with 30 November sowing, mulch reduced the irrigation amount by more than 50 mm in only 35% of years with an average yield loss of 0.3 t ha^−1^. On both soils, the effect of mulch on average irrigation requirement of the 15 October sown crops was very small, although in a few years one extra irrigation was required due to longer crop duration with mulch.

Although the ability of APSIM to simulate the effect of mulch on yield and water balance components is reasonably good, this currently requires separate crop coefficients, determined empirically, for the mulched and non-mulched crops. The model requires improvement to account for the effects of soil temperature modifications (e.g. as a result of mulch) on crop development and growth. The ability to simulate water interception (as a result of irrigation or rainfall) by surface residues, and its subsequent loss by evaporation, is also needed.

### 4.4 Effect of irrigation scheduling on yield and components of the water balance

**Fig. 8:**
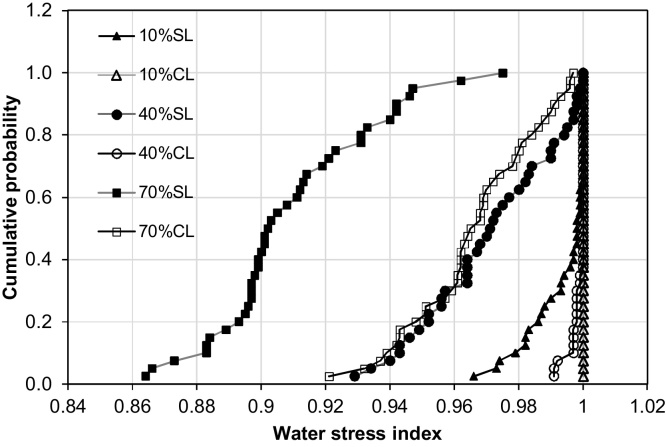
Effect of soil type and irrigation threshold on average Water Stress Index (SL-sandy loam, CL-clay loam) (Scenario 3).

Average grain yields were higher on the clay loam than the sandy loam within the same irrigation schedule (Table 5), and were more stable as irrigation frequency decreased, due to the higher PAWC of the clay loam than the sandy loam (Arora and Gajri, 1998). As irrigation frequency decreased, greater water stress developed on the sandy loam than on the clay loam (Fig. 8). On the clay loam, there was virtually no water deficit stress for schedules from 10 to 40% SWD. However, on the sandy loam, even the 10% SWD schedule resulted in small levels of water deficit stress in many years, although the stress was insignificant. The results are consistent with other findings of lower wheat yields on coarse textured soils than finer textured soils in central Punjab, using the same irrigation and other management practices (Jalota et al., 2006, Yadvinder-Singh et al., 2009).

The higher grain yields on the clay loam soil than on the sandy loam were associated with higher ET. Jalota and Prihar (1998) reported that with adequate soil moisture, evaporation from bare soil and crop ET are higher in finer textured soils. Jalota et al. (2006) found higher ET for wheat on a clay loam soil than on a sandy loam soil under various irrigation treatments. In our study, ET on the sandy loam was decreased from 447 to 197 mm as irrigation frequency decreased from 10% SWD to rainfed, whereas on the clay loam ET was higher, decreasing from 511 to 260 mm. In our simulations, a high proportion of the reduction in ET with reduction in irrigation frequency on the sandy loam came from reduction in T, which led to reduced biomass, while there was no significant decrease in T on the clay loam soil. The higher Es and T on the clay loam soil may be due higher transmission rate (unsaturated hydraulic conductivity) than in the sandy loam (Jalota and Prihar, 1986). Similar results to ours were reported in the field and modelling studies of Jalota et al. (2006) and Arora and Gajri (1998), respectively.

The lower ET on the sandy loam soil is also consistent with the lower amount of irrigation water applied, especially with less frequent irrigation, as reduced biomass production would result in lower soil water depletion. WP_ET_ on the sandy loam was higher than on the clay loam in all irrigation treatments from 10 to 60% SWD, due to lower ET, which more than compensated for the lower yield on the sandy loam. In the field study of Jalota et al. (2006), WP_ET_ was also higher on a sandy loam than on a clay loam. WP_I_ was also higher on the sandy loam soil at each irrigation level.

On the sandy loam soil, maximum WP_ET_ occurred at 50% SWD, similar to the findings from the field experiments of Singh et al. (1980). Similarly, Behera and Panda (2009) observed that grain yield was not reduced in sandy loam soil as irrigation scheduling changed from 10 to 40%SWD, and that WP_ET_ was higher with 40%SWD irrigation scheduling than with 10 and 60% SWD.

### 4.5 Interaction between mulch and irrigation scheduling on components of the water balance and yield

With practical irrigation thresholds of around 40–50% SWD, mulch reduced the number or irrigations by one in approximately 50% of years. This is consistent with the results of field experiments which also show that where irrigation is scheduled according to soil water status, mulch sometimes reduces the number of irrigations by one (Balwinder-Singh et al., 2011c, Naveen-Gupta et al., 2016), and sometimes it does not (Yadvinder-Singh et al., 2008, Naveen-Gupta et al., 2016). For farmers to fully benefit from the potential reduction in the number of irrigations with mulch, practical guidelines or tools to assist them to schedule irrigations based on soil water status are needed.

Mulch reduced the average irrigation amount, however, the effect declined with decreasing irrigation frequency and was generally small. The irrigation water reduction was associated with reduced Es. The average reduction in Es on the clay loam with 40–50% SWD irrigation scheduling was about 30 mm, similar to the reductions of 35–40 mm in the field experiments of Balwinder-Singh et al. (2011b) on a clay loam soil. Within irrigation treatment, the simulated reduction in Es with mulch was always slightly larger on the clay loam, consistent with the findings of the laboratory studies of Gill and Jalota (1996) and Prihar et al. (1996). In the simulations, this was because of the higher stage 1 Es coefficient (12 mm) for the clay loam than the sandy loam (10 mm), together with the fact that surface mulch reduces Es mainly by supressing stage 1 Es (Bond and Willis, 1970). The reduced irrigation amount with mulch led to slightly higher average WP_I_, consistent with findings of Balwinder-Singh et al. (2011c).

The very small increase in simulated grain yield with mulch was probably partly due to increased availability of water for transpiration as a result of reduced Es, as suggested by Zhang et al. (2005). Total crop water use (ET) was reduced by mulch at all irrigation levels due to reduced Es, more so with frequent irrigation. This is in contrast with the field studies of Lascano et al. (1994) and Balwinder-Singh et al. (2011b) who found that there was no effect of mulch on ET. In these studies, water saved from suppressing Es was fully used in T. In contrast, Chakraborty et al. (2008) and Yunusa et al. (1994) reported significantly lower ET of the mulched crops; however, their yields were also lower with mulch, suggesting that the lower ET was at least partly a result of poorer crop growth and reduced T.

## 5 Conclusions

The simulations suggested that, with practical irrigation scheduling (at 50% soil water deficit, SWD), the optimum sowing date for non-mulched wheat in this region is late October to early November on a sandy loam soil, and about one week later on a clay loam, in terms of maximising yield, WP_I_ and WP_ET_. The simulations also suggested that, with mulch, the optimum sowing window is about one week earlier within each soil type. For crops sown at or prior to the optimum time, mulch increased average yield by 0.0–1.3 t ha^−1^, with larger increases as sowing was advanced. On the other hand, when sowing was delayed to mid (sandy loam) or late (clay loam) November, the probability of yield loss with mulch increased, with mean losses of 0.2–0.3 t ha^−1^. The results suggest that the optimum sowing time depends on both soil type and presence or absence of mulch. Further evaluation of the interaction between mulch and sowing date is needed, using both field and simulation experiments, because of the practical importance of knowing the optimum sowing date, how early mulched wheat can be sown without yield loss, and under what circumstances mulching of late sown wheat is detrimental to yield.

On both the sandy loam and clay loam soils, irrigation was highly beneficial in terms of increasing yield and WP_ET_ compared with rainfed wheat, with and without mulch. Grain yield and irrigation input increased with increasing irrigation frequency, more so on the sandy loam. On the latter soil, yield, WP_ET_ and WP_I_ were all maximised with irrigations scheduled at 40–50% SWD. However, on the clay loam, there were trade-offs between yield, WP_ET_ and WP_I_, with yield maximised when irrigations were scheduled at 10–50% SWD, while WP_ET_ and WP_I_ were maximised at 70% SWD. Mulch had very little effect on the thresholds at which each of these parameters were maximised. With irrigations scheduled at 40–50% SWD, mulch reduced the number of irrigations by one in about 50% of years on both soils.

Thus, for irrigated wheat sown at the optimum time in north-west India and with well-managed irrigations based on soil water deficit, mulch is beneficial in terms of reducing irrigation input while increasing yield and WP_ET_. However, for farmers to fully benefit from the potential irrigation reductions with mulch, practical guidelines or tools to assist them to schedule irrigations based on soil water status are needed.

## Figures and Tables

**Table 1:** Mean monthly daily maximum and minimum temperatures and solar radiation, and mean monthly total rainfall and pan evaporation, during the wheat growing season between 1970 and 2010 at Ludhiana, India.

	Temperature (°C)		Radiation (MJ m^−2^ d^−1^)	Rainfall (mm)	Pan evaporation (mm)
	Max.	Min.			
November	26.6	13.4	10.5	7.3 (0–85)^a^	91.5
December	20.0	10.4	6.2	16.5 (0–112)	39.0
January	17.6	10.8	5.4	28.4 (0–83)	47.6
February	18.8	12.5	6.8	32.4 (0–108)	64.4
March	27.3	18.1	12.0	22.6 (0–80)	127.3
April	32.3	21.4	16.4	16.5 (0–122)	227.2

a Values in parenthesis are the range.

**Table 2:** Physical properties of the clay loam and sandy loam soils used in the simulations.

	Sand (%)	Silt (%)	Clay (%)	LL (cm^3^ cm^−3^)	DUL (cm^3^ cm^−3^)	SAT (cm^3^ cm^−3^)	BD (g cm^−3^)	SWCON^a^	KL
Clay Loam									
0–15	46.0	21.3	32.7	0.10	0.31	0.35	1.50	0.50	0.08
15–30	24.0	34.7	41.2	0.12	0.32	0.39	1.71	0.30	0.06
30–60	15.4	39.4	45.1	0.11	0.33	0.38	1.46	0.40	0.04
60–90	40.0	21.5	38.6	0.09	0.31	0.41	1.48	0.50	0.02
90–120	66.6	17.1	15.8	0.07	0.24	0.40	1.33	0.50	0.01
120–150	89.5	6.2	4.3	0.05	0.20	0.38	1.39	0.50	0.01
150–180	89.5	6.5	4.2	0.05	0.20	0.38	1.42	0.50	0.00

Sandy Loam									
0–15	65.6	17.2	17.2	0.07	0.26	0.36	1.61	0.50	0.07
15–30	67.3	17.4	15.3	0.07	0.27	0.31	1.76	0.50	0.06
30–60	71.4	12.0	16.6	0.06	0.23	0.36	1.61	0.50	0.06
60–90	72.2	13.0	14.8	0.06	0.21	0.39	1.53	0.50	0.03
90–120	73.8	12.2	14.0	0.07	0.21	0.39	1.53	0.50	0.02
120–150	80.9	10.9	8.2	0.05	0.21	0.39	1.52	0.50	0.01
150–180	88.1	5.3	8.6	0.05	0.20	0.39	1.52	0.50	0.01

a SWCON is the proportion of soil water above DUL that drains in one day, LL—volumetric water content at wheat crop lower limit. DUL—volumetric water content at drained upper limit. SAT—volumetric water content at saturation. BD—bulk density, KL—water extraction efficiency.

**Table 3:** Effects of sowing date on grain yield (t ha^−1^) and WP_ET_ (kg ha^−1^ mm^−1^) with water non-limiting, and on yield, irrigation amount (mm), WP_ET_ (kg ha^−1^ mm^−1^) and WP_I_ (kg ha^−1^ mm^−1^) with irrigation scheduling at 50% soil water deficit (SWD) on sandy loam and clay loam soils (Scenario 1).

Sowing date	10-Oct	20-Oct	30-Oct	10-Nov	20-Nov	30-Nov	10-Dec	20-Dec	30-Dec
Unlimited water (potential yield conditions)									
Grain yield	2.9	4.3	5.5	6.3	6.4	5.8	5.0	4.4	3.7
WP_ET_	6.3	8.7	10.9	11.9	11.9	11.1	9.8	8.6	7.5

Sandy loam soil- irrigated at 50%SWD									
Grain yield	2.7	3.9	5.2	5.6	5.0	4.2	3.5	2.9	2.5
Irrigation	171	238	269	283	275	265	253	250	227
WP_ET_	11.0	14.1	15.6	14.8	12.9	11.5	10.1	8.8	7.5
WP_I_	15.8	16.4	19.3	19.8	18.2	15.8	13.8	11.6	11.0

Clay loam soil-irrigated at 50% SWD									
Grain yield	2.9	4.2	5.5	6.3	6.4	5.8	5.0	4.4	3.7
Irrigation	160	212	251	311	319	290	274	260	235
WP_ET_	10.8	14.0	15.1	14.8	13.6	12.1	10.6	9.8	8.1
WP_I_	18.2	19.8	21.9	20.3	20.1	20.0	18.2	16.9	15.7

**Table 4:** Grain yield and difference between values for mulched and non-mulched wheat for grain yield (kg ha^−1^), irrigation water input (mm) and crop ET (mm) under different sowing dates for sandy loam and clay loam soils (Scenario 2). Values are means over 40 years of weather data.

Sowing date	15Oct	23Oct	31Oct	7Nov	15Nov	23Nov	30Nov
Sandy Loam							
Yield (kg ha^−1^)							
Non-mulch	3610	4700	5270	5380	5070	4595	4100
Mulch	4860	5700	5800	5400	4800	4300	3770

Difference (Mulch-Non-mulch)							
Yield	1250	1000	530	28	−270	−300	−325
Irrigation	−3	−6	−11	−12	−20	−25	−32
ET	+5	−1.5	−7	−16	−23	−26	−28

Clay Loam							
Yield (kg ha^−1^)							
Non-mulch	3640	4780	5600	6010	6060	5710	5250
Mulch	4930	6000	6460	6530	6050	5510	4940

Difference (Mulch-Non-mulch)							
Yield	1200	1200	900	520	−10	−200	−300
Irrigation	−10	−2	0	−8	−15	−22	−22
ET	−14	−15	−17	−27	−36	−41	−50

**Table 5:** Simulated effect of irrigation and mulch treatments on grain yield, components of the water balance and water productivity for sandy loam and clay loam soils. Values are means over 40 years of weather data. The percentage figures refer to Soil Water Deficit irrigation trigger level, dSWC = difference in profile soil water content between sowing and harvest (harvest minus sowing), WP = water productivity, M = mulch, NM = non-mulch (Scenario 3).

	Sandy loam								Clay loam							
	10%	20%	30%	40%	50%	60%	70%	rainfed	10%	20%	30%	40%	50%	60%	70%	rainfed
Grain yield (t ha^−1^)																
NM	6.00	5.90	5.80	5.70	5.30	4.70	3.90	2.40	6.00	6.00	6.00	6.00	6.00	5.80	5.60	3.20
M	6.40	6.40	6.30	6.00	5.60	4.90	4.00	2.70	6.50	6.50	6.50	6.50	6.50	6.30	6.00	3.50

ET (mm)																
NM	467	432	409	387	361	322	274	199	506	458	430	421	414	402	381	250
M	444	420	394	379	348	313	265	199	457	437	420	415	407	394	370	250

Irrigation (mm)																
NM	426	377	340	306	272	210	148	–	465	391	346	314	295	269	233	–
M	404	370	332	290	250	192	141	–	410	374	329	312	293	261	224	–

Es (mm)																
NM	152	121	105	95	90	85	80	67	185	137	109	101	98	95	94	76
M	103	84	67	61	60	59	58	50	104	85	68	66	64	63	62	55

Transpiration (mm)																
NM	315	310	303	284	269	233	192	132	321	321	321	320	315	306	287	173
M	341	336	327	312	289	254	207	150	353	353	353	350	344	332	309	196

Drainage (mm)																
NM	50	41	36	28	23	16	10	6	47	33	21	13	9	8	7	5
M	54	48	42	30	24	16	13	8	46	35	21	18	13	9	9	6

dSWC (mm)																
NM	+16	+10	+4	−1	−4	−21	−27	−97	+14	+2	−3	−17	−25	−38	−52	−151
M	+15	+11	+5	−4	−11	−25	−25	−96	+13	+7	−7	−15	−21	−36	−50	−150

WP_ET_ (kg ha^−1^ mm^−1^)																
NM	12.8	13.8	14.3	14.7	14.8	14.6	14.2	12.0	11.9	13.1	14.0	14.3	14.5	14.7	14.9	12.9
M	14.5	15.2	15.4	16.2	16.2	15.5	14.7	13.1	14.3	14.9	15.5	15.7	15.9	16.0	16.3	14.0

WP_I_ (kg ha^−1^ mm^−1^)																
NM	14.0	15.8	17.2	18.6	19.7	22.5	26.1	–	12.9	15.4	17.4	19.1	20.3	21.9	24.3	–
M	15.9	17.2	18.9	20.9	22.5	25.3	27.5	–	15.9	17.5	18.8	20.8	22.0	24.2	26.8	–

Irrigation number																
NM	27	13	8	6	4	3	2	–	25	13	8	5	4	3	2	–
M	25	12	7	5	4	3	2	–	24	12	7	5	4	3	2	–

**Table 6:** Weather conditions during keycrop stages of the highest and lowest yielding wheat seasons (Scenario 1).

	Average (40 years) (1970–2010)				Max. yield (8.5 t ha^−1^) year (1988–89)				Min. yield (3.0 t ha^−1^) year (1976–77)			
	Max T (°C)	Min T (°C)	Radn (MJ m^−2^ d^−1^)	PTQ (MJ m^−2^ d^−1^ C^−1^)	Max T (°C)	Min T (°C)	Radn (MJ m^−2^ d^−1^)	PTQ (MJ m^−2^ d^−1^ °C^−1^)	MaxT (°C)	MinT (°C)	Radn (MJ m^−2^ d^−1^)	PTQ (MJ m^−2^ d^−1^ °C^−1^)
Sowing-tillering	21.4	7.4	11.0	–	21.4	7.8	12.1	–	21.6	6.3	11.4	–
Tillering-anthesis	18.8	6.8	12.7	1.53	18.5	5.6	15.4	2.02	19.5	6.8	6.8	0.78
Anthesis-maturity	27.3	12.0	18.4	–	27.1	12.1	15.4	–	30.8	12.3	12.3	–

**Table 7:** Total number of days during which the crop was exposed to more than 34 °C during grain filling period under different sowing dates and mulch conditions during 40 crop seasons (Scenario 2).

Sowing date	Residue condition	Number of days with temperature higher than 34 °C
15Oct	Mulch	1*(1)*^a^
	Non-mulch	1*(1)*
23Oct	Mulch	19 *(8)*
	Non-mulch	9 *(7)*
30Oct	Mulch	54 *(16)*
	Non-mulch	30 *(10)*
7Nov	Mulch	185*(35)*
	Non-mulch	111*(28)*
15Nov	Mulch	367*(38)*
	Non-mulch	265*(40)*

a Values in parenthesis show the number of years out of total 40 years when the crop was exposed to temperature >34 °C.

## References

[bib0005] Aggarwal P.K., Bandyopadhyay S.K., Pathak H., Kalra N., Chander S., Kumar S. (2000). Analysis of yield trends of the rice-wheat system in north-western India. Outlook Agric..

[bib0010] Al-Khatib K., Paulsen G.M. (1984). Mode of high temperature injury to wheat during grain development. Physiol. Plant.

[bib0015] Ambast S.K., Tyagi N.K., Raul S.K. (2006). Management of declining groundwater in the trans indo-Gangetic plain (India): some options. Agric. Water Manage..

[bib0020] Arora V.K., Gajri P.R. (1998). Evaluation of crop growth-water balance model for analysing wheat responses to climate and water-limited environments. Field Crops Res..

[bib0025] Arora V.K., Singh H., Singh B. (2007). Analyzing wheat productivity responses to climatic, irrigation and fertilizer-nitrogen regimes in a semi-arid sub-tropical environment using the CERES-Wheat model. Agric. Water Manage..

[bib0030] Asseng A., Keating B.A., Fillery I.R.P., Gregory P.J., Bowden J.W., Turner N.C., Palta J.A., Abrecht D.G. (1998). Performance of the APSIM-wheat model in western Australia. Field Crops Res..

[bib0035] Balwinder-Singh, Gaydon D.S., Humphreys E., Eberbach P.L. (2011). Evaluating the performance of APSIM for irrigated wheat in Punjab, India. Field Crops Res..

[bib0040] Balwinder-Singh, Eberbach P.L., Humphreys E., Kukal S.S. (2011). The effect of rice straw mulch on evapotranspiration, transpiration and soil evaporation of irrigated wheat in Punjab, India. A gric. Water Manage..

[bib0045] Balwinder-Singh, Humphreys E., Eberbach P.L., Katupitiya A., Singh Yadvinder, Kukal S.S. (2011). Growth, yield and water productivity of zero till wheat as affected by rice straw mulch and irrigation schedule. Field Crops Res..

[bib0050] Balwinder-Singh, Humphreys E., Yadav Sudhir, Gaydon D.S. (2015). Options for increasing the productivity of the rice-wheat system of north-west India while reducing groundwater depletion. Part 1. Rice variety duration, sowing date and inclusion of mungbean. Fields Crops Res..

[bib0055] Behera S.K., Panda R.K. (2009). Effect of fertilization and irrigation schedule on water and fertilizer solute transport for wheat crop in a sub-humid sub-tropical region. Agric. Ecosys. Environ..

[bib0060] Bijay-Singh, Shan Y.H., Johnson-Beebout S.E., Yadvinder S., Buresh R.J., Donald L.S. (2008). Crop residue management for lowland rice-based cropping systems in Asia. Adv. Agron..

[bib0065] Bond J.J., Willis W.O. (1970). Soil Water Evaporation: Ist stage drying as influenced by surface residue and evaporation potential. Soil Sci. Soc. Am. Pro..

[bib0070] Chakraborty D., Nagarajan S., Aggarwal P., Gupta V.K., Tomar R.K., Garg R.N., Sahoo R.N., Sarkar A., Chopra U.K., Sarma K.S.S., Kalra N. (2008). Effect of mulching on soil and plant water status: and the growth and yield of wheat (*Triticum aestivum* L.) in a semi-arid environment. Agric. Water Manage..

[bib0075] Fischer R.A. (1985). Number of kernels in wheat crops and the influence of solar radiation and temperature. J. Agric. Sci. Camb..

[bib0080] Fischer R.A. (2007). Understanding the physiological basis of yield potential in wheat. J. Agric. Sci..

[bib0085] Gill B.S., Jalota S.K. (1996). Evaporation from soil in relation to residue rate, mixing depth, soil texture and evaporativity. Soil Technol..

[bib0090] Gupta P.K., Sahai S., Singh N., Dixit C.K., Singh D.P., Sharma C., Tiwari M.K., Gupta R.K., Garg S.C. (2004). Residue burning in rice-wheat cropping system: causes and implications. Curr. Sci..

[bib0095] Holzworth D.P., Huth N.I., deVoil P.G., Zurcher E.J., Herrmann N.I., McLean G., Chenu K., van Oosterom E., Snow V., Murphy C., Moore A.D., Brown H., Whish J.P.M., Verrall S., Fainges J., Bell L.W., Peake A.S., Poulton P.L., Hochman Z., Thorburn P.J., Gaydon D.S., Dalgliesh N.P., Rodriguez D., Cox H., Chapman S., Doherty A., Teixeira E., Sharp J., Cichota R., Vogeler I., Li F.Y., Wang E., Hammer G.L., Robertson M.J., Dimes J., Carberry P.S., Hargreaves J.N.G., MacLeod N., McDonald C., Harsdorf J., Wedgwood S., Keating B.A. (2014). APSIM—evolution towards a new generation of agricultural systems simulation. Environ. Modell. Softw..

[bib0100] Humphreys E., Kukal S.S., Christen E.W., Hira G.S., Balwinder-Singh Sudhir-Yadav Sharma R.K. (2010). Halting the groundwater decline in north west India—which crop technologies will be winners?. Adv. Agron..

[bib0105] Jalota S.K., Arora V.K. (2002). Model-based assessment of water balance components under different cropping systems in north-west India. Agricultural Water Manage..

[bib0110] Jalota S.K., Prihar S.S. (1986). Effects of atmospheric evaporativity, soil type and redistribution time on evaporation from bare soil. Aust. J. Soil Res..

[bib0115] Jalota S.K., Prihar S.S. (1998). Reducing Soil Water Evaporation with Tillage and Straw Mulching.

[bib0120] Jalota S.K., Sood A., Chahal G.B.S., Choudhury B.U. (2006). Crop water productivity of cotton (*Gossypium hirsutum* L.)-wheat (*Triticum aestivum* L.) system as influenced by deficit irrigation, soil texture and precipitation. Agric. Water Manage..

[bib0125] Jones C.A., Kiniry J.R. (1986). CERES-Maize: A Simulation Model of Maize Growth and Development.

[bib0130] Keating B.A., Carberry P.S., Hammer G.L., Probert M.E., Robertson M.J., Holzworth D., Huth N.I., Hargreaves J.N.G., Meinke H., Hochman Z., McLean G., Verberg K., Snow V., Dimes J.P., Silburn M., Wang E., Brown S., Bristow K.L., Asseng S., Chapman S., McCown R.L., Freebairn D.M., Smith C.J. (2003). An overview of APSIM, a model designed for farming systems simulation. Eur. J. Agron..

[bib0135] Ladha J.K., Pathak H., Gupta R.K. (2007). Sustainability of the rice-wheat cropping system: issues, constraints, and remedial options. J. Crop Improv..

[bib0140] Lascano R.J., Baumhardt R.L., Hicks S.K., Heilman J.L. (1994). Soil and plant water evaporation from strip-tilled cotton—measurement and simulation. Agron. J..

[bib0145] Naveen-Gupta, Yadav Sudhir, Humphreys E., Kukal S.S., Balwinder-Singh P.L., Eberbach P.L. (2016). Effects of tillage and mulch on the growth, yield and irrigation water productivity of a dry seeded rice-wheat cropping system in north-west India. Field Crops Res..

[bib0150] Ortiz-monasterio J.I., Dhillon S.S., Fischer R.A. (1994). Date of sowing effects on grain-yield and yield components of irrigated spring wheat cultivars and relationships with radiation and temperature in Ludhiana, India. Field Crops Res..

[bib0155] Pathak H., Wassmann R. (2009). Quantitative evaluation of climatic variability and risks for wheat yield in India. Clim. Change.

[bib0160] Pathak H., Ladha J.K., Aggarwal P.K., Peng S., Das S., Singh Y., Singh B., Kamra S.K., Mishra B., Sastri A., Aggarwal H.P., Das D.K., Gupta R.K. (2003). Trends of climatic potential and on-farm yields of rice and wheat in the Indo-Gangetic Plains. Field Crops Res..

[bib0165] Porter J.R., Gawith M. (1999). Temperatures and the growth and development of wheat: a review. Eur. J. Agron..

[bib0170] Prihar S.S., Jalota S.K., Steiner J.L. (1996). Residue management for reducing evaporation in relation to soil type and evaporativity. Soil Use Manage..

[bib0175] Probert M.E., Keating B.A., Thompson J.P., Parton W.J. (1995). Modelling water, nitrogen, and crop yield for a long-term fallow management experiment. Aust. J. Exp. Agric..

[bib0180] Probert M.E., Dimes J.P., Keating B.A., Dalal R.C., Strong W.M. (1998). APSIM's water and nitrogen modules and simulation of the dynamics of water and nitrogen in fallow systems. Agric. Sys..

[bib0185] Randhawa A.S., Dhillon S.S., Singh W. (1981). Productivity of wheat varieties, as influenced by the time of sowing. J. Res. (PAU).

[bib0190] Ritchie J.T., Godwin D.C., Otter S. (1985). CERES-Wheat: A User-oriented Wheat Yield Model. Preliminary Documentation: AGRISTARS Publication No. YM-U3-04442-JSC-18892.

[bib0195] Sidhu H.S., Manpreet S., Humphreys E., Yadvinder S., Balwinder S., Dhillon S.S., Blackwell J., Bector V., Malkeet S., Sarbjeet S. (2007). The Happy Seeder enables direct drilling of wheat into rice stubble. Aust. J. Exp. Agric..

[bib0200] Sidhu, H.S., Manpreet-Singh, Blackwell, J., Humphreys, E., Bector, V., Yadvinder-Singh, Malkeet-Singh, Sarbjit-Singh, 2008. Development of the Happy Seeder for direct drilling into combine harvested rice. In: Humphreys, E., Roth, C.H. (Eds.), Permanent Beds and rice-residue management for rice-wheat systems in the Indo-Gangetic Plain. Proceedings of a workshop held at PAU, Ludhiana, India from 7 to 9 September 2006. ACIAR Proceedings No. 127. pp. 159–170. Available at http://www.aciar.gov.au/publication/term/18.

[bib0205] Sidhu H.S., Manpreet S., Yadvinder S., Blackwell J., Lohan S.K., Humphreys E., Jat M.L., Vicky Singh Sarbjeet S. (2015). Development and evaluation of the Turbo Happy Seeder for sowing wheat into heavy rice residues in NW India. Field Crops Res..

[bib0210] Singh G., Singh P.N., Bhushan L.S. (1980). Water use and wheat yields in northern India under different irrigation regimes. Agric. Water Manage..

[bib0215] Thorburn P.J., Probert M.E., Robertson F.A. (2001). Modelling decomposition of sugarcane surface residues with APSIM-Residue. Field Crops Res..

[bib0220] Timsina J., Godwin D., Humphreys E., Yadvinder S., Bijay S., Kukal S.S., Smith D. (2008). Evaluation of options for increasing yield and water productivity of wheat in Punjab, India using the DSSAT-CSM-CERES-Wheat model. Agric. Water Manage..

[bib0225] Wang E., Robertson M.J., Hammer G.L., Carberry P.S., Holzworth D., Meinke H., Chapman S.C., Hargreaves J.N.G., Huth N.I., McLean G. (2003). Development of a generic crop model template in the cropping system model APSIM. Eur. J. Agron..

[bib0230] Yadvinder-Singh, Bijay-Singh, Ladha J.K., Khind C.S., Khera T.S., Bueno C.S. (2004). Effects of residue decomposition on productivity and soil fertility in rice-wheat rotation. Soil Sci. Soc. Am. J..

[bib0235] Yadvinder-Singh, Bijay-Singh, Timsina J. (2005). Crop residue management for nutrient cycling and improving soil productivity in rice-based cropping systems in the tropics. Adv. Agron..

[bib0240] Yadvinder-Singh, Sidhu, H.S., Manpreet-Singh, Humphreys, E., Kukal, S.S. and Brar, N.K. 2008. Straw mulch, irrigation water and fertiliser N management effects on yield, water use and N use efficiency of wheat sown after rice. In: Humphreys, E., Roth, C.H. (Eds.), Permanent Beds and rice-residue management for rice-wheat systems in the Indo-Gangetic Plain. Proceedings of a workshop held at PAU, Ludhiana, India from 7 to 9 September 2006. ACIAR Proceedings No. 127, pp. 171–181. Available at http://www.aciar.gov.au/publication/term/18.

[bib0245] Yadvinder-Singh, Humphreys E., Kukal S.S., Singh B., Kaur A., Thaman S., Prashar A., Yadav S., Timsina J., Dhillon S.S., Kaur N., Smith D.J., Gajri P.R. (2009). Crop performance in permanent raised bed rice-wheat cropping system in Punjab, India. Field Crops Res..

[bib0250] Yunusa I.A.M., Sedgley R.H., Siddique K.M.H. (1994). Influence of mulching on the pattern of growth and water-use by spring wheat and moisture storage on a fine textured soil. Plant Soil.

[bib0255] Zhang X., Chen S., Liu M., Pei D., Sun H. (2005). Improved water use efficiency associated with cultivars and agronomic management in the North China Plain. Agron. J..

[bib0260] Zhao H., Dai T., Jing Q., Jiang D., Cao W. (2007). Leaf senescence and grain filling affected by post-anthesis high temperatures in two difference wheat cultivars. Plant Growth Regul..

